# What Was I Thinking? Eye-Tracking Experiments Underscore the Bias that Architecture Exerts on Nuclear Grading in Prostate Cancer

**DOI:** 10.1371/journal.pone.0038023

**Published:** 2012-05-30

**Authors:** Dario Bombari, Braulio Mora, Stephan C. Schaefer, Fred W. Mast, Hans-Anton Lehr

**Affiliations:** 1 Institute of Psychology, University of Bern, Bern, Switzerland; 2 University Institute of Pathology, Centre Hospitalier Universitaire Vaudois, Lausanne, Switzerland; University of Campinas, Brazil

## Abstract

We previously reported that nuclear grade assignment of prostate carcinomas is subject to a cognitive bias induced by the tumor architecture. Here, we asked whether this bias is mediated by the non-conscious selection of nuclei that “match the expectation” induced by the inadvertent glance at the tumor architecture. 20 pathologists were asked to grade nuclei in high power fields of 20 prostate carcinomas displayed on a computer screen. Unknown to the pathologists, each carcinoma was shown twice, once before a background of a low grade, tubule-rich carcinoma and once before the background of a high grade, solid carcinoma. Eye tracking allowed to identify which nuclei the pathologists fixated during the 8 second projection period. For all 20 pathologists, nuclear grade assignment was significantly biased by tumor architecture. Pathologists tended to fixate on bigger, darker, and more irregular nuclei when those were projected before kigh grade, solid carcinomas than before low grade, tubule-rich carcinomas (*and vice versa*). However, the morphometric differences of the selected nuclei accounted for only 11% of the architecture-induced bias, suggesting that it can only to a small part be explained by the unconscious fixation on nuclei that “match the expectation”. In conclusion, selection of « matching nuclei » represents an unconscious effort to vindicate the gravitation of nuclear grades towards the tumor architecture.

## Introduction

*Intellectus humanus in iis quae semel placuerunt – aut quia recepta sunt et credita, aut quia delectant – alia etiam omnia trahit ad suffragationem et consensum cum illis: et licet major sit instantiarum vis et copia, quae occurrunt in contrarium: tamen eas aut non observat, aut contemnit, aut distinguendo summovet et rejicit, non sine magno et pernicioso praejudio, quo prioribus illis syllepsibus auctoritas maneat inviolata. In: Francisci Baconi NOVUM ORGANUM, sive indicia de interpretatione naturae (1620)*

(The human understanding, when it has developed an opinion – either as being the received opinion or as being aggreeable to itself – draws all things else to support and agree with it. And though there be a greater number and weight of instances to be found on the other side, yet these it either neglects and despises, or else by some distinction sets aside and rejects; in order that this great and pernicious predetermination the authority of its former conclusion may remain inviolate. *In: Francis Bacon NOVUM ORGANUM, 1620*).

A pathologist translates an image that he/she sees under a microscope into a diagnosis. In the case of a malignant tumor, clinicians not only expect a name for the tumor, but also a variety of prognostic and predictive features that will help to choose the right treatment and to counsel the patient. Obviously, the act of interpreting microscope slides depends on ample knowledge and year–long experience. However, what is less well recognized is that this act of slide interpretation is also subject to complex biases, expectations, and confounding factors that risk to modify the final conclusion [1]–[3].

In a recent study, we have shown that the architectural growth pattern of a prostate cancer, which provides the morphological basis for the time-honored Gleason grade [4], induces a powerful bias in the mind of pathologists that affects the subsequent assignment of a nuclear grade. The nuclear grade describes the degree of atypia of tumor nuclei, where small, pale and round nuclei connote minimal atypia (grade 1) and large, dark and angulated nuclei marked atypia (grade 3). While architectural and nuclear grades shared a strong prognostic impact, the prognostic power of the nuclear grade was lost entirely when nuclei were graded out of their architectural context [2], suggesting that the nuclear grade “borrows” its prognostic impact from the architectural grade. In an effort to better understand the mechanisms that underlie this powerful confirmation bias, we asked whether pathologists « unconsciously » search and analyze those tumor cells that match their expectation. In order to experimentally address this question, we have now used eye-tracking technology, asking 12 board-certified pathologists and 8 pathology residents to assign nuclear grades to prostate cancer images displayed on a computer screen.

## Methods

### Participants

12 board certified pathologists and 8 pathology residents, all of whom working at the university institute of pathology, CHUV, Lausanne.

### Stimuli

**Figure 1:**
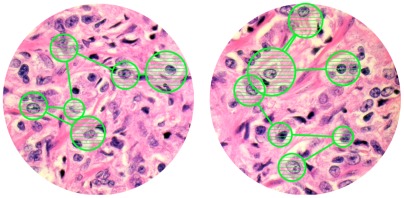
Eye-tracking scan paths of fixated nuclei. The high resolution computer screen, on which the above described slide presentation was displayed, was interfaced with an eye-tracking device, which allowed to follow the eye movements of the pathologists and hence to record exactly which nuclei they looked at and for how long. Scan paths and attention maps were computed with the aid of SMI BeGaze Analysis software. All nuclei that were fixated for a minimum of 100 milliseconds were recorded. The hatched green circles depict the location of the fixation within the HPF and the size of the circle the duration of the fixation. The lines between the circles depict the scan path. All nuclei that were selected by the eyetracking program were later analyzed by Photoshop-based image analysis in terms of nuclear size, hyperchromasia, heterochromasia and roundness.

**Figure 2:**
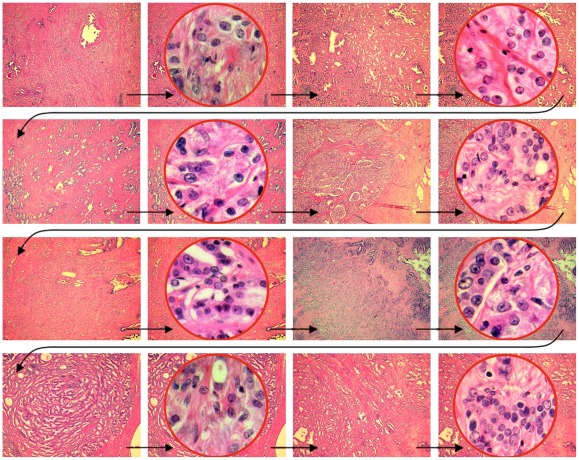
Slide show of HPFs before different architectural backgrounds. A slide presentation was displayed on a 24″ computer screen that depicted HPFs of 20 different prostate carcinomas in central round windows before the background of a low power image of the tumor architecture. What the pathologists did not know was that from each of the 20 prostate carcinomas, two HPFs were shown, once before a low power image with well-formed tubular structures (corresponding to a combined Gleason grade 2–3) and again some time later before a low power image showing solid tumor architecture (corresponding to a combined Gleason grade 4–5). The presentation was automatically timed to show each slide fir exactly 8 seconds. Pathologists were asked to assign nuclear grades for each HPF. This part of the presentation lasted for a little over 10 minutes. Immediately after that, 40 slides were shown that depicted 10 random nuclei from each of the displayed HPFs, arranged in a 2×5 matrix (see figure 2).

Prostate carcinomas were selected from the archive of the institute of pathology. The study protocol was accepted by the institutonal ethical review board (CEP–VD N°BB15–2008). Digital images taken from microscope slides were used in a strictly codified fashion without any patient identifiers and the procedures were in accordance with the Helsinki Declaration of 1975, revised in 1983. Circular high power fields (« HPF ») were photographed from 20 different prostate carcinomas. For that purpose, images are taken using an Olympus C4040 camera attached via a C-mount to an Olympus BX45 microscope at 20x magnification, creating a jpg file (with minimal file compression) of 2272×1704 pixels (size 11.1 M), which was then cropped to a circular field (8.3 M), corresponding to the microscope field of a 40× objective, with a diameter of 1704 pixels (circle size 25 cm, respolution 180dpi, figure 1). In the same way, low power images were taken from 40 different prostate carcinomas at 4x magnification (2272×1704 pixels, size 11.1 M, then cropped to 2272×1550 pixels, size 10.1 M). In a random fashion, HPFs of each of 20 carcinomas were shown on a 24″ screen once immediately following a low power image of a low-grade carcinoma showing small regular tubules, corresponding to a combined Gleason grade of 2–3 (such an architectural image reflects a high degree of tumor differentiation and implies slow tumor growth) and once again – in a random fashion – following a low power image of another carcinoma characterized by solid or unorderly tumor growth, corresponding to a combined Gleason grade of 4–5 (the absence of regular tubule formation reflects advanced tumor dedifferentiation and implies aggressive tumor biology). The order of the screen shots is shown in figure 2. In order to make sure that there was no difference in terms of nuclear atypia of the two random HPFs from each of the 20 prostate carcinoma, we analyzed by nuclear morphometry (see below) that the nuclei in the two respective HPFs were identical, showing no differences in terms of size, hyperchromasia, heterochromasia nor roundness.

### Eye Tracking

The high-resolution computer screen, on which the images were displayed, was attached to a iView X RED eyetracking device (SMI, Berlin, Germany), which allowed to register the pathologists’ eye fixations. This permitted to document which nuclei they looked at and for how long. The iView X RED eyetracker has a gaze position accuracy of 0.4°, which is important for visually complex stimuli, and a sampling rate (corneal reflex and pupil diameter) of 50 Hz. The iView X RED eyetracker was interfaced with a PC, thus allowing for time logged presentation of visual stimuli. A major advantage of iView RED is that there is no head mount. Pathologists were hence free to move their head without being constantly reminded that their eye fixations were being monitored. We used Experiment Center software (SMI, Berlin) for presentation of the stimuli and iViewX (SMI, Berlin) for eye movement data acquisition. Results were exported in open format (.txt) and analyzed using SPSS and Excel. Scan paths and attention maps were computed with the aid of SMI BeGaze Analysis software (version 2.5 SMI, Berlin, see figure 1).

### Experimental procedure

**Figure 3:**
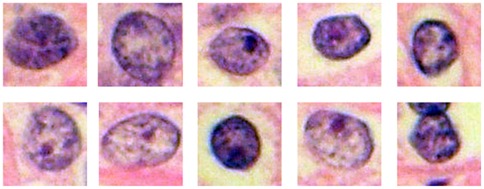
10 random nuclei isolated out of their architectural context. Ten nuclei were randomly selected from each HPF displayed in the first part of the presentation (see figure 2), yet this time isolated out of their architectural context, and displayed in an orderly arranged 2×5 matrix on the computer screen for a duration of 8 seconds. Pathologists were asked to assign nuclear grades for each group of 10 nuclei. Later, these same nuclei were analyzed by Photoshop-based image analysis in terms of nuclear size, hyperchromasia, heterochromasia and roundness of the nuclear contour.

Participants were tested individually. They were seated in front of a 24″ high-resolution computer monitor, set up in a quiet room in the institute of pathology, allowing pathologists to perform the experiments within the realm of their usual working environment. Prior to the experiment proper, a short calibration procedure had to be performed, during which each pathologist was asked to follow with his/her eyes the course of a small point on the black computer screen. Eye movements were recorded throughout the entire experiment. During the experiment, a total of 80 microscopic images were displayed on the screen, alternating between low power images and HPFs of prostate carcinomas. HPFs were displayed in a circle superimposed on the low power image (Figure 2). Each image was displayed for 8 seconds. While the HPFs were presented, the pathologists were asked to grade the nuclei and speak out loud the nuclear grade that he/she would assign. Intermediate grades (1.5 and 2.5) were explicitly allowed). No guidance was given as to what constitutes a grade 1, a grade 2, or a grade 3 nucleus, and none of the participating pathologists asked instructions concerning that point. These grades were noted and the experimenter used a response button to document the time point of the announcement. At the end of this presentation, which lasted for a little over 10 minutes, the participants were shown a series of 40 images (8 seconds, each), which displayed ten randomly selected nuclei from each of the 40 HPFs used in the first part of the presentation, but this time cropped out of their architectural context and displayed simultaneously arranged in a 2×5 matrix (figure 3). The cropped nuclei were transferred into the 2×5 matrix in the same image resolution of 180dpi and the same size as they were on the initial high power field.

### Nuclear morphometry

Image analysis was performed in analogy to previously published methods [2]. High–resolution HPF images (8.1 M) were opened in Photoshop (version CS2, Adobe Systems Inc., San Jose, CA). All nuclei displayed in the circular areas were numbered, isolated using the lasso tool and a computer pen on a high-resolution touch-sensitive computer screen (Cintiq 15x, Wacom, Taiwan), and exported using copy-paste into separate Photoshop files. The histogram command in the image menu was then selected to document for each nucleus the following parameters: (i) the number of pixels, reflecting the mean nuclear size, (ii) the mean greylevel as a measure of nuclear hyperchromasia, (iii) the standard deviation of the greylevel histogram as a measure of nuclear heterochromasia, and (iv) using the shape filter in a commercially available Photoshop plug-in (The Image Processing Tool Kit, Version 2, Reindeer Games, Ashville, NC), the form factor as a measure of the roundness of the nuclear contour (values ranging from 1 to values around 0.6 for entirely round or highly angulated, “unround” nuclei, respectively). The exact methods for nuclear morphometry have been described in detail [2]. The data were imported into an Excel file and used for the calculation of nuclear characteristics of those nuclei that every pathologist had fixated according to the scan paths of each image (figures 1). In the same way, we quantified the nuclear characteristics of the 10 random nuclei displayed in the 2×5 matrix (figure 3) in order to perform a linear regression analysis, thus identifying nuclear morphometric feature(s) each individual pathologist applied during nuclear grade assignment.

## Results

**Figure 4:**
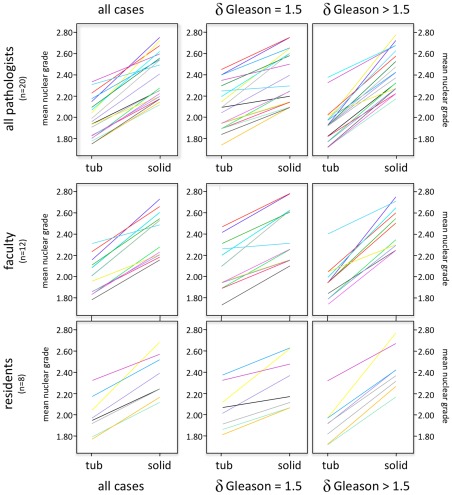
Nuclear grades assigned to prostate carcinomas are biased by the architectural growth pattern. The y-axes of the nine graphs depict the nuclear grades assigned by each of 20 pathologists on HPFs that were displayed on a computer screen for 8 seconds. Each line shows the two grades assigned by one pathologist for the same HPF depicted before a low grade architecture, rich in tubules (“tub”) and a high grade, solid architecture (“solid”). Note that for every pathologist, lower nuclear grades were assigned when the HPFs were depicted before a tubule-rich carcinoma and higher nuclear grades when the HPFs were presented before a solid carcinoma. The left panels show data for all 20 pathologists (upper panels), for 12 board-certified pathologists (“faculty”, middle panel) and for 8 residents (lower panel). The right three panels show the data calculated on those pairs of HPFs where the background images differed by more than 1.5 Gleason points and the middle panels the data ro those HPFs where the background images differed by only 1.5 Gleason points.

We analyzed the mean nuclear grades assigned by the participants for HPFs presented after low power images of either low grade or high grade carcinomas. We found that, without a single exception, all of the 20 participating pathologists systematically assigned lower nuclear grades to HPFs that were displayed in the context of an architecturally low grade carcinoma, i.e. shown immediately after the low power image of a Gleason grade 2–3 carcinoma (mean = 2.02; SD = 0.18). In contrast, systematically higher nuclear grades were assigned to the HPFs of the same crcinoma, yet displayed in the context of a Gleason grade 4–5 carcinoma (mean 2.42; SD = 0.20). These striking differences, which confirm pilot observations on three pathologists reported previously [2], are statistically highly significant in the analysis of the group of 20 pathologists (2–tailed paired samples test, t(19) = 15.50, p <0.001, figure 4, upper left panel). The extent of the nuclear grade bias induced by the tumor architecture depended on the difference in architectural differentiation of the two respective low power images for each HPF: the bias was more pronounced when the two low power images differed by more than 1.5 Gleason points (1.94±0.18 vs. 2.47±0.20) than if they differed by only 1.5 Gleason points (2.09±0.22 vs. 2.37±0.22, figure 4, middle and right upper panels, t(19) = 8.43, p <0.001). This t-test was calculated by comparing participants' delta for nuclear grade in the condition where the two low power images differed by only 1.5 Gleason points and when the differences was more than 1.5.

We then investigated whether the nuclear grade bias induced by the tumor architecture depended on the experience of the pathologist. To this end, we divided the group of pathologists into residents and board certified pathologists (faculty). We found that the extent of the architectural bias was comparable between the two groups of pathologists and did hence not appear to depend on the years of experience (residents: 2.02±1.8 vs. 2.42±0.21; faculty: 2.02±0.24 vs. 2.39±0.21, figure 4 left lower panels, t(19) = 0.657, p = 0.52). In a more refined analysis, we found no difference between four junior residents in the first two years of their training and four more advanced residents, or between four recently board certified pathologists and eight pathologists with many years of experience (*F*(3,16) = 0.197, *MSE* = 0.016, *p* = 0.90). Also, we found no difference when we compared those eight board-certified pathologists who routinely read prostate cancers and those four who have not or only exceptionally been confronted with prostate pathology for years (*F*(1,18) = 1.746, *MSE* = 0.013, *p* = 0.20).

By performing Spearman correlations between assigned nuclear grades and nuclear morphometric features assessed by image analysis of the 10 nuclei per HPF that were displayed in the 2×5 matrix (Figure 3; defining a significant correlation with rho values >3 and p values <05), we found that for 9 of the pathologists, nuclear grades correlated solely with nuclear size (pixel numbers), for 1 pathologist solely with hyperchromasia (mean greylevel values), for 2 pathologists with both roundness and nuclear size, and for 3 pathologists with both roundness and hyperchromasia. Not a single pathologist based his nuclear grade assignments on nuclear heterochromasia. For 5 pathologists, we failed to identify any correlation between nuclear grades and any of the four tested nuclear morphometric features.

**Figure 5:**
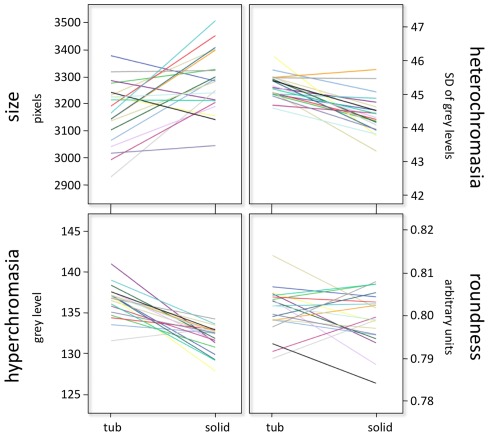
The architectural bias influences how pathologists select nuclei (eye-tracking experiments). Nuclear morphometric features of nuclei selected during eye-tracking experiments. Each line shows the morphometric features for nuclei selected by one pathologist for HPFs of each case then displayed either before a low grade architecture, rich in tubules (“tub”) and a high grade, solid architecture (“solid”). Note that for every pathologist, larger (size, upper left panel), darker (hyperchromasia, lower left panel), and coarser nuclei (heterochromasia, upper right panels) were fixated when the HPFs were shown before a solid carcinoma. The differences were statistically significant at the P<0.01 level (size) and the P<0.001 level (chromasia). In contrast, nuclear selection appeared not to be based on nuclear roundness (lower right panel, not significant).

We next asked whether the architectural background image affected the selection of nuclei that are fixated in the HPFs. For that purpose, we identified the nuclei that each pathologist had fixated for a minimum of 100 milliseconds during the eight second viewing period and computed their nuclear morphometric features (size, hyperchromasia, heterochromasia, roundness). As expected, pathologists looked at different nuclei depending on the architectural background image that immediately preceded its presentation, with a clear tendency to fixate smaller, paler nuclei when the HPF was displayed following a low power image of a low grade carcinoma rich in tubules and on larger, darker nuclei when the HPF of the same case was displayed after a low power image of a high gradecarcinoma (figure 5, upper left panel). The difference was statistically highly significant for the nuclear morphometric parameters size (3172±110 vs. 3283±109 pixels, t(19), = 3.52; p <0.01, figure 5 left upper panel), hyperchromasia (136.2±2.0 vs. 131.5±1.6 arbitrary units, t(19),  = 7.57; p <0.001, figure 5, left lower panel) and heterochromasia (45.24±0.35 vs. 44.41±0.56; *t*(19) = 6.12, *p* <0.001, figure 5, right upper panel), whereas no effect was found for roundness (*t*(19) = 1.15, *p* = 0.26, figure 5, right lower panel). For the parameter size and hyperchromasia, the selection of different nuclei was even more pronounced when the difference in architectural differentiation between the background images was larger (>1.5 Gleason points: nuclear size: *t*(19) = 5.84, *p* <0.001; hyperchromasia: *t*(19) = 5.28, *p* <0.001, data not shown).

We then asked whether the magnitude of the grade bias induced by the tumor architecture could be explained by this selection of nuclei that pathologists fixated. We established for each pathologist simple linear regressions between the nuclear grades that he/she had given to the nuclei displayed in the 2×5 matrix (figure 3) and the mean nuclear morphometric features of these 10 nuclei. We selected for each pathologist the morphometric feature that showed the strongest correlation (Spearman) with his/her nuclear grade (see above). Based on the slope of this regression, we then calculated which difference in nuclear grade would have resulted solely from nuclear selection under the influence of the tumor architecture (figure 5). This allowed to identify for each pathologist to which extent his/her selection of nuclei contributed to the overall nuclear grade bias induced by the tumor architecture. We found that the visual selection of the nuclei accounted for hardly more than one tenth of the overall bias (mean value 11.1%, median value 8.5%, range 0%–44.0%) of the difference in nuclear grades subjectively assigned to HPFs when displayed after either low grade or high grade background images.

## Discussion

The principal observations of this study are (i) that the assignment of nuclear grades is inadvertently biased by the architectural growth pattern of a given tumor, (ii) that pathologists look at different nuclei under the influence of the architecture-induced bias, actively searching for nuclei that “match” the architectural grade, but (iii) that the magnitude of the architecture-induced bias can only to a small fraction be explained by the unconscious selection of matching nuclei.

The first observation confirms, albeit on a more robust scale and with high statistical power, a prior observation by our group [2]. That prior study was initially intended to simply compare the prognostic power of architectural and nuclear grading in 183 prostate carcinomas. To our surprise, we had found that the nuclear grades that were assigned by each of the three pathologists were significantly linked with the architectural grades of the tumors (Gleason grades) and were only to a small extent based on true nuclear morphometric features. Of particular importance was the observation that while the nuclear grade predicted tumor progression just as strongly as did the architectural grade, the prognostic power of the nuclear grade was lost when nuclei were graded out of their architectural context in a 5×2 matrix similar to the one that we have used in our present study [2]. Apparently, the architectural pattern acts as a powerful cue to which the nuclear grade assignment inevitably gravitates. This may be explained by a well-known phenomenon that has been described in cognitive psychology as “confirmation bias”. This concept understands the tendency for people to search and selectively marshal information that confirms a tentatively held hypothesis and not seek, or even discard, information that support an opposite conclusion (see the quote by Francis Bacon at the beginning of the manuscript). In order to accept that a bias can be operative in such a highly reproducible fashion – affecting each of 20 participating pathologists – we need to acknowledge that histological diagnostic is an intuitive process during which heuristic processes are at work. If one considers that scanning a single histological slide at high magnification contains information that equals a storage capacity of about 1 GB of hard disk space, a purely analytical approach to slide viewing would rapidly overpower our limited cognitive resources. Also, no histological slide is identical with another and, as MacLendon pointed out, “one must open one’s eye and mind to the information to be gleaned from each new slide” [1], limiting our possibility to scrutinize slides in a purely analytical fashion. Heuristics is a powerful tool, which navigates pathologists through the diagnostic work-up process and renders this process highly effective [5]. As it is the case for many other situations, we accomplish this task by unconsciously employing mental short-cuts [5], [6]. Charlin and coworkers have applied these principles to medical diagnostics by introducing the concept of “disease scripts” [7]. With increasing experience, clinicians encapsulate patho-physiological concepts, acquired knowledge, and the experience gained on prior patients/cases into complex scripts that are then applied to efficiently handle new, but “similar” situations. These authors proposed that during the diagnostic work-up, disease scripts are activated by initial cues/primes and then guide the selection and interpretation of further information in the context of the assumed disease. For each attribute in a script, the value with the greatest probability of occurrence is set as the default value and this default values is maintained unless it is actively rejected [7], [8].

In the next part of our study, we aimed to better understand the mechanisms behind the selection of nuclei that are in synchrony with the architectural pattern. By tracking the pathologists' eye movements, we addressed the question whether we unconsciously search and select those nuclei that match best the expectation that is induced by the architecture. We found that pathologists tended to look at larger or darker nuclei when HPFs were displayed before a high grade, solid architecture and vice versa (figure 5). When applying the concept of disease scripts to our observations, we propose that architecture could function as cue to activate the “high grade prostate cancer” script, where an assumed high nuclear grade then guides visual search processes towards large, hyperchromatic nuclei. Since pathologists will find at least some larger, darker nuclei among the total number of nuclei in the HPF, their expectation is sufficiently confirmed and there is no convincing reason to reject the default value “high grade nuclear grade”. Several authors have postulated that – if different options are offered – our mind tends to “satisfice” with the matching information [9] and to simply ignore those nuclei that would not be consistent with the default nuclear grade. In this context, it is noteworthy that the cognitive bias in assigning nuclear grades was just as pronounced in junior residents as it was in older residents, young faculty, or experienced pathologists (figure 4). Our results are hence in line with the work by Chimowith and coworkers, who observed that senior neurologists made less mistakes than residents, with the exception of those mistakes that were due to cognitive biases (notably due to “satisficing” with information that matched the working hypothesis), which was just as frequently seen in experienced neurologists as it was seen in residents [10].

Interestingly, the visual selection of nuclei (figure 5) could not account for the extent of the difference in nuclear grade assignments (figure 4), which was an order of magnitude bigger. It is conceivable that the architectural bias may lead to an increased perception of subtle differences in nuclear morphology. In other words, a slightly larger, slightly darker nucleus may be perceived as substantially larger and substantially darker when the solid architectural background biases the pathologists. Alternatively, our mind may have decided for a nuclear grade even before sending the eyes toward the nuclei at the “matching” end of the spectrum of nuclear atypias available within the displayed HPF. Hedonistic psychology holds that our minds are more comfortable with observations that are in harmony with a held hypothesis then with evidence that rejects a hypothesis [11]. Nuclear selection may hence serve the sole purpose of confirming a preconceived decision and hence to vindicate the gravitation of the nuclear grades towards the tumor architecture. As efficient and economical as heuristic reasoning may be, it is inherently flawed and taking shortcuts comes at the price of occasional and predictive errors [12], [13]. This study illustrates one such error in the realm of diagnostics in pathology.

Our findings are also relevant in the light of the current discussion about errors in medicine [13]–[15] and in particular about “error culture” and personal responsibility [16], [17]. If it is true, as the father of the Swiss cheese model of human error, James Reason stated [18] that “our propensity for certain types of error is the price we pay for the brain's remarkable ability to think and act intuitively”, does this relieve us of the responsibility of diagnostic errors that are the consequence of such biases? In the case of prostate carcinoma, the good news is that the described systematic error in assigning “biased” nuclear grades is of no adverse consequence for the patients [2], as architecture is a time-honored powerful prognostic parameter [4]. Yet, can the same statement be held up for other malignant tumors? In the case of breast cancer, for instance, architecture and nuclear features contribute equally to tumor grading [19] and the assigned tumor grade is up to this day the key element on which chemotherapy decisions are based on. Evidently, the bias can manifest itself in tumor gradings, and we should ask the question whether it can – in one way or the other – be controlled or suppressed? It has been speculated that medical students who are taught in the new case-based curriculum are less susceptible to cognitive biases, since they tend to construct diagnostic cases in a bottom-up fashion, but effective ways to teach students how to avoid the pitfalls of using heuristics have yet to be identified [20]. Standard operating procedures and the consequent application of rules may be effective in minimizinig errors due to cognitive biases [13] – albeit possibly at the price of efficacy. One such rule is applied by some pathologists who categorically refuse to take into account the clinical context of a biopsy (and the clinician’s expectation that frequently transpires from its description) before taking an unbiased look at the histological slides. This rule usually originates from personal (negative) experience or from “gut feeling”. To our knowledge, not a single scientific study has ever investigated whether – and to what extent – these expectations communicated by the clinicians influence the diagnosic work of the pathologist.

A wealth of knowledge about biases has been ascertained in the field of cognitive psychology [9], [21], in particular as applied to law enforcement, marketing, biomedical research, military strategy or the stock market [22]–[25], but also to clinical medicine [3], [6], [12], [26], [27]. Illustrative examples for cognitive biases induced by affective expectations are the powerful placebo effects [27] or the observation that the identical Californian Cabernet Sauvignon tastes significantly better when served out of a $90 bottle then out of a $10 bottle [22]. Until now, however, these concepts have not been considered of relevance for the work of a diagnostic pathologist. Eyetracking experiments such as the ones performed in this study and by others [28] may open the way to novel insights into the way how pathologists view slides and arrive at diagnostic conclusions. We believe that the results from this study showing that architecture induces a strong bias on subsequent nuclear grade assignment, are relevant for a better understanding of how pathologists operate during their diagnostic activity. Reflecting on our “mode of operation” and identifying putative sources of biases are first steps to develop effective counter-measures to avoid that these modes of operation translate into clinically relevant errors. In other words: asking “how am I thinking” may eventually help to avoid having to ask “what was I thinking?” when reviewing slides after erroneous diagnostic decisions.
